# MAG‐DPA curbs inflammatory biomarkers and pharmacological reactivity in cytokine‐triggered hyperresponsive airway models

**DOI:** 10.1002/prp2.263

**Published:** 2016-10-18

**Authors:** Rayan Khaddaj‐Mallat, Roddy Hiram, Chantal Sirois, Marco Sirois, Edmond Rizcallah, Sofia Marouan, Caroline Morin, Éric Rousseau

**Affiliations:** ^1^ Faculty of Medicine and Health SciencesDepartment of Obstetrics‐GynecologyUniversité de SherbrookeSherbrookeQCJ1H 5N4Canada; ^2^ Faculty of Medicine and Health SciencesService of Thoracic SurgeryUniversité de SherbrookeSherbrookeQCJ1H 5N4Canada; ^3^ Faculty of Medicine and Health SciencesDepartment of PathologyUniversité de SherbrookeSherbrookeQCJ1H 5N4Canada

**Keywords:** Airway hyperresponsiveness, docosapentaenoic acid, human bronchi, monoacylglyceride, resolution of inflammation

## Abstract

Bronchial inflammation contributes to a sustained elevation of airway hyperresponsiveness (AHR) in asthma. Conversely, omega‐3 fatty acid derivatives have been shown to resolve inflammation in various tissues. Thus, the effects of docosapentaenoic acid monoacylglyceride (MAG‐DPA) were assessed on inflammatory markers and reactivity of human distal bronchi as well as in a cultured model of guinea pig tracheal rings. Human bronchi were dissected and cultured for 48 h with 10 ng/mL TNF‐*α* or IL‐13. Guinea pig tracheas were maintained in organ culture for 72 h which was previously shown to trigger spontaneous AHR. All tissues were treated with increasing concentrations of MAG‐DPA (0.1, 0.3, and 1 *μ*mol/L). Pharmacomechanical reactivity, Ca^2+^ sensitivity, and western blot analysis for specific phosphoproteins and transcription factors were performed to assess the effects of both cytokines, alone or in combination with MAG‐DPA, on human and guinea pig airway preparations. Although 0.1 *μ*mol/L MAG‐DPA did not significantly reduce inflammatory biomarkers, the higher concentrations of MAG‐DPA (0.3 and 1 *μ*mol/L) blunted the activation of the TNF‐*α*/NF *κ*B pathway and abolished COX‐2 expression in human and guinea pig tissues. Moreover, 0.3 and 1 *μ*mol/L MAG‐DPA consistently decreased the Ca^2+^ sensitivity and pharmacological reactivity of cultured bronchial explants. Furthermore, in human bronchi, IL‐13‐stimulated phosphorylation of CPI‐17 was reversed by 1 *μ*mol/L MAG‐DPA. This effect was further amplified in the presence of 100 *μ*mol/L aspirin. MAG‐DPA mediates antiphlogistic effects by increasing the resolution of inflammation, while resetting Ca^2+^ sensitivity and contractile reactivity.

**Abbreviations**
AHR: airway hyperresponsiveness
ASM: airway smooth muscle
CPI‐17: protein kinase C‐dependent phosphorylation of the 17 kDa myosin phosphatase inhibitor protein
HB: human bronchi
MAG‐DPA: docosapentaenoic acid monoacylglyceride
PUFA: polyunsaturated fatty acid
TNF‐*α*: tumor necrosis factor alpha

## Introduction

Chronic asthma is distinguished from milder variants by increased airway inflammation and hyperresponsiveness (Murdoch and Lloyd 2010; Olin and Wechsler 2014). It has been shown that polyunsaturated fatty acids (PUFA) may have beneficial effects on various cell types (Goldman et al. 1983). However, *n*−3 PUFA levels are decreased in airway illnesses which are characterized by excess airway inflammation and enhanced n6‐PUFA, including arachidonic acid derivatives, as in the case of asthma (Connor 2000) and cystic fibrosis (Freedman et al. 2004; Fredman and Serhan 2011).

Appropriate experimental models in respiratory pathophysiology and pharmacology research aimed at analyzing the mechanical properties and signaling pathways involved in airway hyperresponsiveness (AHR) are limited (Morin et al. 2005; Chiba et al. 2010). Studies using bronchial preparations and isolated cultured cells have shown that inflammatory mediators and cytokines (TNF‐*α*, IL‐13, and/or IL‐1*β*) can alter Ca^2+^ homeostasis in ASM and render cells nonspecifically overreactive to pharmacological agonists (Hunter et al. 2003; Tliba et al. 2003; Yang et al. 2004). Numerous biochemical stimuli – methacholine via M3 receptors, histamine via the H1 receptor, and U‐46619 via TP receptors – lead to the activation of various signaling pathways, controlling ASM contraction (Hall 2000; Léguillette and Lauzon 2008; Sanderson et al. 2008). Hence, the PKC/CPI‐17 signaling pathway have been involved in the control of airway smooth muscle (ASM) tone (Morin and Rousseau 2006), with abnormalities in PKC‐dependent signaling being associated with respiratory diseases including COPD (Zhang et al. 2004). The higher the CPI‐17 phosphorylation level, the higher the Ca^2+^ sensitivity which in turn results in an increased tone (Sakai et al. 2005). Finally, the TNF‐*α*‐induced increase in the Ca^2+^ sensitivity of MLC_20_ phosphorylation is achieved through stimulation of RhoA/Rho‐kinase pathway leading to inhibition of myosin light‐chain phosphatase (MLCP) (Hunter et al. 2003).

Several groups have reported beneficial effects of omega‐3 polyunsaturated fatty acids in this setting. Docosahexaenoic acid (DHA, 22:6*n*−3), eicosapentaenoic acid (EPA, 20:5*n*−3), and docosapentaenoic acid (DPA, 22:5*n*−3) have been shown to prevent autoimmune diseases (Simopoulos 2002) and to possess anti‐inflammatory properties (Connor 2000; Giudetti and Cagnazzo 2012; Dalli et al. 2013). Distinct studies have demonstrated that *n*−3 PUFAs in monoacylglyceride form are generally recognized as safe in the food industry. These compounds are well absorbed by the gastrointestinal tract with no toxicity (Fortin 2009, 2012). The monoacylglyceride of EPA was moreover shown to increase the bioavailability of EPA compared to commercially available marine oil (Cruz‐Hernandez et al. 2012). Consistent with these observations, new omega‐3 sn1‐monoacylglycerides were recently synthesized (Fortin 2012). MAG‐EPA was shown to target cytoplasmic signaling pathways to reverse short‐term AHR in guinea pig tracheal tissues (Khaddaj‐Mallat and Rousseau 2015a), hence MAG‐EPA was able to abolish the airway hyperresponsivenes in an in vivo model of allergic asthma (Morin et al. 2012a,b). MAG‐DHA and one of its metabolite, RvD1, were able to reverse the IL‐13‐induced TNF‐*α*/NFxB signaling pathway in human distal bronchi (Khaddaj‐Mallat et al. 2015b). The rational and relevance of using guinea pig airways relies on the homology in respiratory physiology and pharmacological reactivity between guinea pig and human airways (Morin et al. 2005; Canning and Chou 2008).

Among *n*−3 PUFAs, docosapentaenoic acid (DPA) is an intermediary elongated fatty acid between EPA and DHA (Kaur et al. 2010, 2011a). The beneficial effects of this polyunsaturated fatty acid have been detected in cardiovascular (Rissanen et al. 2000) and neuronal systems (Kim et al. 2003) and liver (Kaur et al. 2011b). However, no report has conclusively assessed the physiological and pharmacological effects of either DPA or MAG‐DPA in human and animal models of AHR. Recently, DPA and DHA monoacylglycerides (MAG‐DPA and MAG‐DHA) were demonstrated to induce apoptosis in colorectal carcinoma cells (HCT116 cells), with MAG‐DPA displaying the higher antiproliferative effects (Morin et al. 2013c). A previous study also demonstrated that oral administration of 230 mg/kg MAG‐DPA in a rat model of pulmonary hypertension decreased NF*ĸ*B and p38 MAPK activation, leading to a reduction in MMP2, MMP9, and VEGF expression levels (Morin et al. 2014).

Our working hypothesis is that a proinflammatory state induces hyperreactivity in human distal bronchi and guinea pig trachea and that MAG‐DPA treatment inhibits cytokine effectors and main inflammatory biomarkers COX‐2, NF*κ*B, and PPAR*γ*. However, PPAR*γ* is a potential target for lung disease and targeting this transcription factor may result in a potential new treatment for chronic inflammatory lung diseases such as asthma and COPD (Christman et al. 2000; Becker et al. 2006). Hence, MAG‐DPA could represent a potentially valuable compound to alleviate airway responsiveness.

The aim of this study was therefore to assess the effects of MAG‐DPA on bronchial inflammatory markers and pharmacologically induced smooth muscle active tone using two well‐described in vitro models, namely, a human bronchi inflammatory model (TNF‐*α* and IL‐13 stimulated) (Morin and Rousseau 2006; Khaddaj‐Mallat et al. 2015b) and a guinea pig tracheal organoid culture model of airway hyperresponsiveness (Morin et al. 2005). Herein, we report the first evidence that the newly synthesized MAG‐DPA displays resolving properties and prevents AHR in lung tissues.

## Materials and Methods

### Drugs and chemical reagents

MAG‐DPA was synthesized and purified by SCF‐Pharma (Rimouski, Quebec, Canada). TNF‐*α*, methacholine chloride (MCh), histamine, aspirin, and *β*‐actin antibodies were purchased from Sigma (St. Louis, MO). U‐46619 as well as TNF‐*α* and COX‐2 antibodies were obtained from Cayman Chemical (Ann Arbor, MI). PPAR*γ* and P‐NF*κ*B antibodies as well as IL‐13 cytokine were purchased from Cell Signaling Technology (Boston, MA), anti‐CPI‐17 and anti‐phospho‐CPI‐17 antibodies were purchased from Cedarlane (Burlington, Ontario, Canada). GPR‐32 antibody was purchased from Abcam (Cambridge, MA). DMEM/F12 and penicillin–streptomycin were obtained from Gibco Invitrogen Corp. (Burlington, Ontario, Canada).

### Culture of human distal bronchi

This study was approved by our Institutional Ethics Committee (Protocol No.: CRC05‐088‐S1‐R6). Consenting patients (*N* = 15) hospitalized for lung carcinoma were recruited and human lung tissues were obtained from the operating room via the Department of Pathology. “Tumor‐free” tissue samples were transported to our laboratory in sterile Krebs' solution, pH 7.4. Distal bronchi samples were dissected under binocular control, and bronchial segments of 4 mm in length and 0.5–0.8 mm in diameter were cultured for 48 h in Dulbecco's modified Eagle's medium: nutrient mixture‐F12 (DMEM‐F12) containing 1% penicillin/streptomycin (Morin et al. 2012a). Human bronchi numbers differ depending on the size of the lung resection (*n* = 10–12/human lung resection). Airways were either untreated (control) or treated with 10 ng/mL TNF‐*α*, TNF‐*α* + 0.1 *μ*mol/L MAG‐DPA, TNF‐*α* + 0.3 *μ*mol/L MAG‐DPA, or TNF‐*α* + 1 *μ*mol/L MAG‐DPA. Other sets of human bronchi were pretreated with 10 ng/mL IL‐13 or IL‐13 combined with 1 *μ*mol/L MAG‐DPA or 1 *μ*mol/L MAG‐DPA + 100 *μ*mol/L acetylsalicylic acid (ASA). All tissues were incubated at 37°C in 5% CO_2_. The *n* values for each experimental condition were reported in the figure legends.

### Tissue preparation and organ culture of guinea pig airways

Male or female albino guinea pigs (Hartley 300–350 g) were anesthetized by intraperitoneal injection of a lethal dose of sodium pentobarbital (40 mg/kg). All procedures involving animal tissues were performed according to Canadian Council for Animal Care (CCAC) guidelines (protocol number 018–12). The trachea was then sectioned into eight tubular segments of equal length and each main bronchus was dissected into two sections. The tissues were used directly after dissection (fresh) or placed in individual wells of a culture plate containing DMEM‐F12 culture medium (Gibco, ref catalog no. 11330032) supplemented with 1% penicillin/streptomycin (Morin et al. 2005). Explants were maintained in culture for 72 h in either untreated (control) or treated (every 24 h, for 72 h) 0.1 *μ*mol/L MAG‐DPA, 0.3 *μ*mol/L MAG‐DPA, or 1 *μ*mol/L MAG‐DPA. Culture plates were placed in a humidified incubator at 37°C under 95% O_2_ and 5% CO_2_ conditions (Khaddaj‐Mallat and Rousseau 2015a).

### Isometric tension measurements

Tension measurements were performed exactly as described previously (Morin et al. 2012a). A basal tension of 0.8 g for human bronchi and 1 g for guinea pig tracheal rings was applied. The pharmacologically induced contractile responses by specific agonists and eicosanoids were assessed on active tensions, using transducer systems coupled to Polyview software (Grass‐Astro‐Med Inc, West Warwick, RI) enabling to perform data acquisition and analysis. The amplitudes of maximal tensions were expressed for a given agonist concentration (1 *μ*mol/L MCh, 1 *μ*mol/L His, or 30 nmol/L U‐46619) or 80 mmol/L KCl for each tested explant (Morin et al. 2012a; Khaddaj‐Mallat and Rousseau 2015a).

### β‐escin permeabilization and Ca^2+^ sensitivity

Human bronchial rings were mounted in organ baths to which a 0.8‐g tension was applied. After measuring the contractile response elicited by 1 *μ*mol/L MCh in Krebs solution, rings were incubated for 25 min in low Ca^2*+*^ relaxing solution (pCa 9) and permeabilized at 25°C with 50 *μ*mol/L *β*‐escin. Tension developed by the permeabilized bronchial rings was measured at 37°C according to free Ca^2+^ concentrations expressed in terms of pCa (pCa = −log [Ca^2+^]). Step increases in free Ca^2+^ (from pCa = 9.0 to 5.3) induced reproducible concentration–response curves to free Ca^2+^ concentrations, indicating successful permeabilization under these conditions (Morin et al. 2012a).

### Preparation of nuclear, cytosolic, and microsomal fractions

Human bronchi and guinea pig tracheal rings were weighed and promptly transferred in a buffer containing (mmol/L): 300 sucrose, 20K‐PIPES, 4K‐EGTA, pH 7.2, and a cocktail of protease (protease inhibitor pellets from Roche Diagnostics, Indianapolis, IN) and phosphatase inhibitors (10 *μ*mol/L Na_2_VO_3_). The latter prevented the dephosphorylation of regulatory proteins. Tissues were homogenized at 4°C and centrifuged at 1500*g* in the same buffer. The nuclear, cytosolic, and microsomal fractions were prepared as described previously (Khaddaj‐Mallat and Rousseau 2015a).

### Isolation of airway smooth muscle cells

Airway smooth muscle cells were isolated as described previously (Pyne and Pyne 1993). Cells were cultured, treated, and corresponding lysates were prepared exactly as described previously (Khaddaj‐Mallat and Rousseau 2015a).

### SDS‐PAGE and western blot analyses

Western blots were performed using antibodies against CPI‐17, P‐CPI‐17, P‐NF*κ*B, GPR‐32, COX‐2, PPAR*γ*, TNF‐*α*, and *β*‐actin proteins. Blot immunostainings were revealed on Kodak film, digitized using a Xerox GPD PS V3.4377.6.0 set at 600 dpi and analyzed using Image J software (Morin et al. 2012a).

### Data analysis and statistics

Results are expressed as means ± SEM with *n* indicating the number of experiments. Statistical analyses were performed using a Student's *t* test or one‐way analysis of variance (ANOVA). Differences were considered statistically significant when *P <* *0.05. Data curve fittings were performed using Sigma Plot 10 (SPSS‐Science, Chicago, IL) to determine EC_50_ values.

## Results

### Effects of TNF‐α and MAG‐DPA on cumulative concentration–response curves

**Figure 1:**
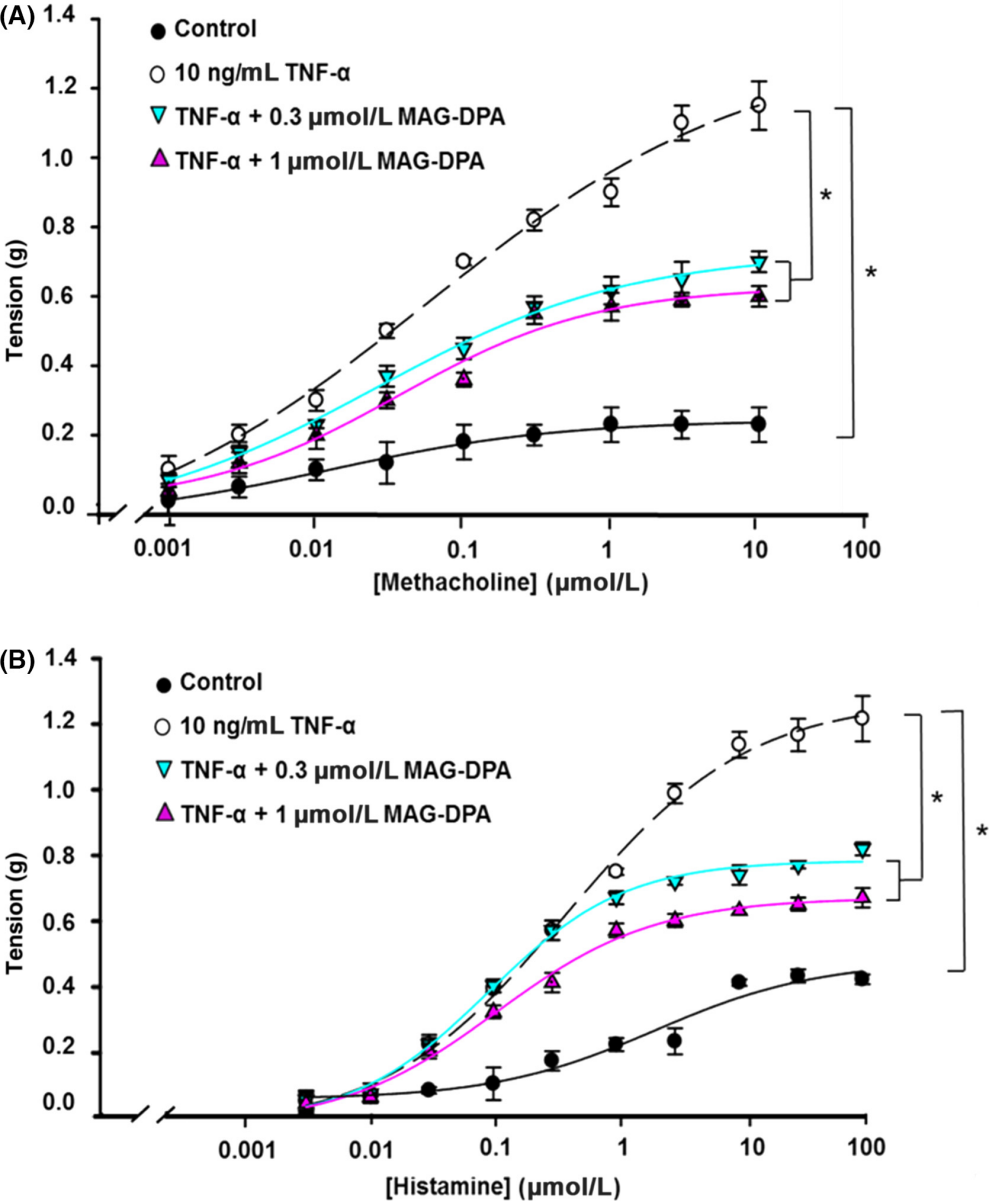
Modulation of the pharmacological reactivities of TNF‐*α*‐pretreated human bronchi (HB) by MAG‐DPA. (A) Methacholine and (B) histamine cumulative concentration–response curves (CCRC) were generated using untreated (control, solid circle), 10 ng/mL TNF‐*α*‐treated bronchi (open circle), TNF‐*α* + 0.3 *μ*mol/L MAG‐DPA (cyan inverted triangle), and TNF‐*α* + 1 *μ*mol/L MAG‐DPA‐treated HB (purple triangle). Tensions are expressed as absolute value (g) and the stats annotations refer to the maximal tensions (MT). Each point represents the mean tonic response ± SEM, with *n* = 12 for each experimental condition (*P ˂ 0.05). In human bronchi, mean maximal tension (MT, g) and EC_50_ values (*μ*mol/L) induced by MCh were MT_control_: 0.23 ± 0.05, EC_50_: 0.09 *μ*mol/L; MT_TNF__‐*α*_: 1.15 ± 0.07, EC_50_: 0.05 *μ*mol/L; MT_TNF__‐*α*+0.3 *μ*mol/L_ _MAG__‐_ _DPA_: 0.70 ± 0.03, EC_50_: 0.055 *μ*mol/L; MT_TNF__‐*α*+1 *μ*mol/L_ _MAG__‐_ _DPA_: 0.60 ± 0.03, EC_50_: 0.065 *μ*mol/L. While for His, MT_control_: 0.40 ± 0.01, EC_50_: 1 *μ*mol/L; MT_TNF__‐*α*_: 1.2 ± 0.07, EC_50_: 0.5 *μ*mol/L; MT_TNF__‐*α*+0.3 *μ*mol/L_ _MAG__‐_ _DPA_: 0.80 ± 0.02, EC_50_: 0.15 *μ*mol/L; MT_TNF__‐*α*+1 *μ*mol/L_ _MAG__‐_ _DPA_: 0.65 ± 0.03, EC_50_: 0.18 *μ*mol/L.

To investigate the pharmacomechanical properties of TNF‐*α* pretreated human bronchi in the absence or presence of MAG‐DPA, cumulative concentration–response curves to pharmacological agonists such as methacholine (MCh) and histamine (His) were measured. Figure 1A depicts the cumulative concentration–response curves (CCRC) to MCh in control and TNF‐*α*‐treated human bronchi in the absence or presence of either 0.3 or 1 *μ*mol/L MAG‐DPA. CCRC demonstrated that 48 h pretreatment with 10 ng/mL TNF‐*α* induced a hyperresponsiveness to MCh with an apparent EC_50_ value (*μ*mol/L) and a mean maximal tension (MT, g) of 0.09 *μ*mol/L and 1.15 ± 0.07 g comparatively to 0.05 *μ*mol/L and 0.23 ± 0.05 g in control conditions. Conversely, treatment with either 0.3 *μ*mol/L or 1 *μ*mol/L MAG‐DPA significantly reduced the mean amplitudes of 0.70 ± 0.03 g and 0.60 ± 0.03 g, respectively.

CCRC to histamine showed that 48 h pretreatment with 10 ng/mL TNF‐*α* significantly increased the responsiveness to this agonist and reduced the apparent EC_50_ value and MT to 0.5 *μ*mol/L and 1.2 ± 0.07 g compared to control conditions (EC_50_ values of 1 *μ*mol/L, MT of 0.40 ± 0.01 g) (Fig. 1B). Combined TNF‐*α* treatment with either 0.3 *μ*mol/L MAG‐DPA or 1 *μ*mol/L MAG‐DPA significantly reduced the contractile response to His on pretreated tissues, with apparent EC_50_ values of 0.15 and 0.18 *μ*mol/L, and MT of 0.80 ± 0.02 g and 0.65 ± 0.03 g, respectively. Together, these data demonstrate that TNF‐*α* treatments increase reactivity of human bronchi to MCh and His, while MAG‐DPA cotreatments largely reverse this overreactivity with limited effects on apparent EC_50_ values for the agonist used.

### Anti‐inflammatory effect of MAG‐DPA on TNF‐α‐pretreated human bronchi

**Figure 2:**
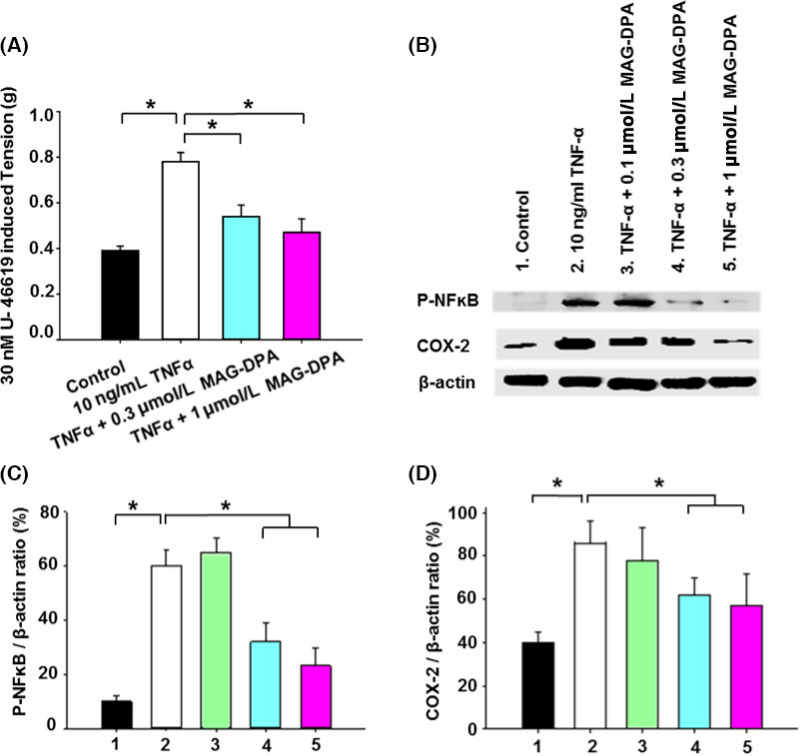
Inhibitory effects of MAG‐DPA on overreactivity response and inflammatory biomarkers in human bronchi. (A) Bar histogram displaying the mean pharmacomechanical responses induced by 30 nmol/L U‐46619 (a thromboxane prostanoid receptor agonist) in control, TNF‐*α*, TNF‐*α *+ 0.3 *μ*mol/L MAG‐DPA, and TNF‐*α *+ 1 *μ*mol/L MAG‐DPA‐treated bronchi (*n* = 12 for each experimental condition, *P ˂ 0.05). (B) Western blot analysis of nuclear protein fractions derived from untreated (control), TNF‐*α*, TNF‐*α *+ 0.1 *μ*mol/L MAG‐DPA, TNF‐*α *+ 0.3 *μ*mol/L MAG‐DPA, and TNF‐*α *+ 1 *μ*mol/L MAG‐DPA‐treated bronchi using specific antibodies against P‐NF *ĸ*B, cyclooxygenase 2 (COX‐2), and *β*‐actin. Note the higher staining level of P‐NF *ĸ*B and COX‐2 in TNF‐*α*‐treated bronchi (lane 2). Quantitative analysis of P‐NF *κ*B (C) and COX‐2 (D) protein detection. Staining densities in the protein fractions were expressed as a function of *β*‐actin signals (*n* = 4, *P <* *0.05).

To assess the putative proresolving effect of MAG‐DPA on pre‐established inflammation, human bronchi were cultured either in the absence (control) or presence of 10 ng/mL TNF‐*α*. TNF‐*α* was also combined with either 0.3 *μ*mol/L MAG‐DPA or 1 *μ*mol/L MAG‐DPA treatments during 48 h and then challenged with 30 nmol/L U‐46619, a TP receptor agonist (Fig. 2A) as this agonist was previously shown to increase the Ca^2+^ sensitivity and tone of human bronchi (Morin et al. 2012a,b b), TNF‐*α* pretreatment consistently increased the reactivity to 30 nmol/L U‐46619 when compared to control conditions. However, MAG‐DPA treatment (0.3 or 1 *μ*mol/L) was able to reverse this overreactivity induced by TNF‐*α*. Hence, despite the presence of TNF‐*α*, the mean agonist‐induced tone in the presence of 1 *μ*mol/L MAG‐DPA was close to the tone developed in control condition. Thus, *μ*M concentrations of MAG‐DPA were able to blunt TNF‐*α*‐induced airway hyperresponsiveness to U‐46619.

To determine the molecular pathways involved in MAG‐DPA anti‐inflammatory effects, protein levels of phospho‐NF*κ*B (P‐NF*κ*B) and COX‐2 were analyzed by western blots in the nuclear fractions of TNF‐*α*‐treated human bronchi. TNF‐*α* treatment resulted in increased detection levels of P‐NF*κ*B and COX‐2 protein when compared to mean levels obtained from control bronchi. MAG‐DPA treatments decreased the phosphorylation level of p65 NF*κ*B and COX‐2 in lung tissues comparatively to the levels observed in TNF‐*α*‐treated tissues (Fig. 2B). Quantitative immunoblot analyses revealed that TNF‐*α* pretreatment resulted in a significant increase in both P‐NF*κ*B/*β*‐actin and COX‐2/*β*‐actin ratios when compared to the ratios measured in control bronchi, whereas significant reductions in these specific ratios were quantified in the presence of all three concentrations of MAG‐DPA (Fig. 2C and D, respectively). Moreover, quantitative analysis revealed a significant decrease in GPR‐32/*β*‐actin ratios in TNF‐*α*‐pretreated human bronchi, whereas 1 *μ*mol/L MAG‐DPA pretreatment significantly increased the expression levels of GPR‐32 immunostaining band (Plate S1A and B).

### Normalization of Ca^2+^ sensitivity to MAG‐DPA treatments

**Figure 3:**
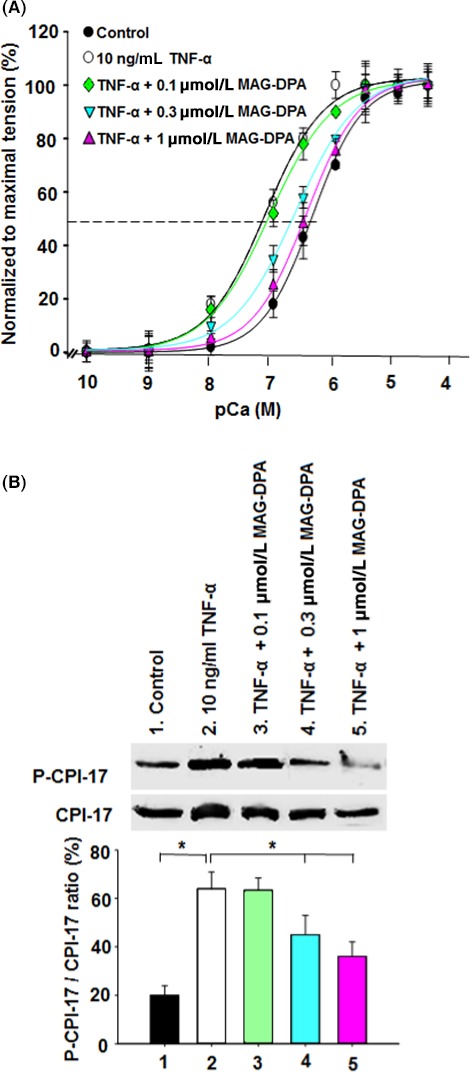
MAG‐DPA modulates Ca^2+^ sensitivity in TNF‐*α*‐pretreated human bronchi. (A) CCRCs to free [Ca^2+^] obtained from *β*‐escin permeabilized tissues in control (solid circles), TNF‐*α* (open circles), TNF‐*α* + 0.1 *μ*mol/L MAG‐DPA (green diamond), TNF‐*α* + 0.3 *μ*mol/L MAG‐DPA (cyan inverted triangle), and TNF‐*α* + 1 *μ*mol/L MAG‐DPA (purple triangle). Each point represents the mean ± SEM, where *n* = 12 for each experimental condition (*P ˂ 0.05). (B) Western blot and quantitative analysis of cytosolic protein fractions derived from control, TNF‐*α*, TNF‐*α* + 0.1 *μ*mol/L MAG‐DPA, TNF‐*α* + 0.3 *μ*mol/L MAG‐DPA and TNF‐*α* + 1 *μ*mol/L MAG‐DPA‐treated human bronchi using specific antibodies against the phosphorylated form of CPI‐17 (P‐CPI‐17) and total CPI‐17. Note the reduced staining of P‐CPI‐17 bands in the presence of 1 *μ*mol/L MAG‐DPA, as opposed to increased staining in TNF‐*α*‐treated bronchi. Staining densities of P‐CPI‐17 in the cytosolic fractions were expressed as a function of CPI‐17 signals. Significant differences were observed between control and TNF‐*α* alone as well as the latter with the higher MAG‐DPA concentrations (0.3 and 1 *μ*mol/L). Results are representative of six similar experiments (*P ˂ 0.05).

To assess the putative inhibitory effects of MAG‐DPA on airway smooth muscle Ca^2+^ sensitivity, specific measurements were performed using *β*‐escin permeabilized bronchial rings. Figure 3A clearly demonstrates that TNF‐*α* treatment induced Ca^2+^ hypersensitivity when compared to Ca^2+^ sensitivity measured in control bronchi (EC_50_ values of 0.10 and 0.65 *μ*mol/L, respectively). MAG‐DPA (0.1, 0.3, and 1 *μ*mol/L) decreased the Ca^2+^ hypersensitivity triggered by TNF‐*α*, with calculated EC_50_ values of 0.15, 0.4, and 0.62 *μ*mol/L, respectively. The Ca^2+^ sensitivity determined in the presence of TNF‐*α* + 1 *μ*mol/L MAG‐DPA moreover reached that observed in control condition (EC_50_ value of 0.63 and 0.65, respectively), thus suggesting that MAG‐DPA is able to completely reverse the effect induced by TNF‐*α*. However, no difference was observed in mean Ca^2+^ sensitivity between TNF‐*α* and TNF‐*α* combined with the lower MAG‐DPA concentration (0.1 *μ*mol/L) in pretreated bronchi (Fig. 3A, dark triangle).

Complementary experiments were performed to assess the putative processes supporting the above negative feedback mechanism induced by MAG‐DPA on this Ca^2+^–tension relationship. The status of the CPI‐17 regulatory protein, known to be involved in modulating the Ca^2+^ sensitivity of the contractile machinery (Sakai et al. 2005; Morin et al. 2012a), was therefore assessed in this bronchial inflammatory model. Bronchial explants were cultured for 48 h in the absence (control) or presence of either 10 ng/mL TNF‐*α* alone or TNF‐*α* combined with either 0.1, 0.3, or 1 *μ*mol/L MAG‐DPA. Western blot and quantitative analyses revealed no significant differences between TNF‐*α* and TNF‐*α* + 0.1 *μ*mol/L MAG‐DPA. However, pretreatment of bronchial explants with either 0.3 or 1 *μ*mol/L MAG‐DPA largely decreased the P‐CPI‐17/CPI‐17 staining density ratio when compared to the ratio in TNF‐*α*‐treated bronchi (Fig. 3B). This result is consistent with the aforementioned data assessing Ca^2+^ sensitivity under corresponding experimental conditions.

### Effects of MAG‐DPA on IL‐13‐induced hyperresponsiveness

**Figure 4:**
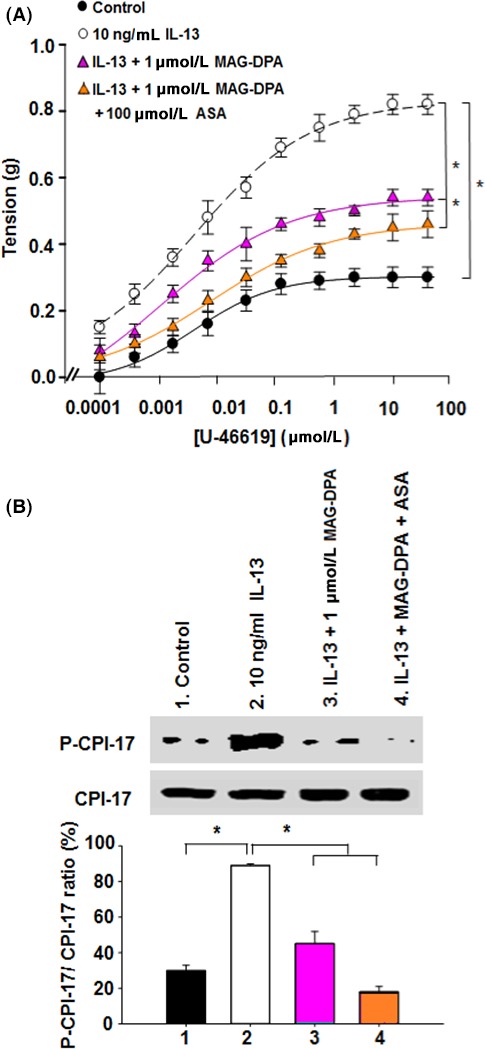
Effect of MAG‐DPA treatment on airway hyperresponsiveness developed by IL‐13‐pretreated human bronchi. (A) CCRC to U‐46619 were performed on control (solid circles), 10 ng/mL IL‐13 (open circles), IL‐13 + 1 *μ*mol/L MAG‐DPA (purple triangle), and IL‐13 + 1 *μ*mol/L MAG‐DPA + 100 *μ*mol/L acetylsalicylic acid (ASA)‐treated bronchi (orange triangle). Tensions are expressed as absolute value (g) and the stats annotations refer to the comparison of the mean maximal tension. Each point represents the mean tonic response ± SEM, with *n* = 12 for each experimental condition (*P ˂ 0.05). MT_control_: 0.30 ± 0.03, EC_50_: 5.5 nmol/L; MT_IL__‐13_: 0.82 ± 0.03, EC_50_: 7 nmol/L; MT_IL__‐13+1 *μ*mol/L_ _MAG__‐_ _DPA_: 0.54 ± 0.02, EC_50_: 5.8 nmol/L; MT _IL__‐13+_ _MAG__‐_ _DPA__+100 *μ*mol/L_ _ASA_: 0.45 ± 0.04, EC_50_:10 nmol/L. (B) Western blot and quantitative analysis of P‐CPI‐17/CPI‐17 density ratios in cytosolic fractions derived from human bronchi obtained from control (untreated), 10 ng/mL IL‐13, IL‐13 + 1 *μ*mol/L MAG‐DPA, IL‐13 + MAG‐DPA + 100 *μ*mol/L ASA, and IL‐13 + 300 nmol/L RvD1‐treated human bronchi. Staining densities of the P‐CPI‐17 immunoreactive bands are expressed as a function of the CPI‐17 signal. Results shown are representative of six similar experiments (*P <* *0.05).

The effect of MAG‐DPA on IL‐13 pretreated human distal bronchi was first assessed on mechanical reactivity. Results revealed that 10 ng/mL IL‐13 treatments induced an overreactivity to 30 nmol/L U‐46619 (with a mean maximal tension of 0.82 ± 0.03 g and an apparent EC_50_ value of 7 nmol/L) when compared to control conditions (mean maximal tension of 0.30 ± 0.03 g and EC_50_ value of 5.5 nmol/L) (Fig. 4A). In contrast to the above pharmacological response to U46619 in the presence of IL‐13, 1 *μ*mol/L MAG‐DPA and 1 *μ*mol/L MAG‐DPA + 100 *μ*mol/L ASA significantly reduced the induced reactivity triggered by the activation of TP receptors (with a mean maximal tension of 0.54 ± 0.02 g, EC_50_: 5.8 nmol/L and 0.45 ± 0.04 g, EC_50_:10 nmol/L, respectively).

Further experiments were performed to ascertain whether or not MAG‐DPA was able to downregulate the phosphorylation level of CPI‐17 protein (Fig. 4B), under the same experimental conditions as described above. Quantitative analysis of CPI‐17 phosphorylation levels in cytosolic fractions of human bronchi revealed a significant decrease in phospho‐CPI‐17/total CPI‐17 ratio in the presence of 1 *μ*mol/L MAG‐DPA as well as MAG‐DPA plus ASA as compared to the enhanced ratio detected upon IL‐13 treatment (Fig. 4B). In order to further investigate the putative processes that support this negative feedback mechanism induced by MAG‐DPA on AHR, complementary experiments were performed to assess the expression status of GPR‐32 receptor. Western blot analysis revealed that 1 *μ*mol/L MAG‐DPA, 1 *μ*mol/L MAG‐DPA + 100 *μ*mol/L ASA, and 300 nmol/L RvD2 pretreatments significantly increased GPR‐32/*β*‐actin staining density ratio when compared to IL‐13 pretreated human bronchi (Plate S2A and B).

### Effect of MAG‐DPA on cultured guinea pig airway active tone

**Figure 5:**
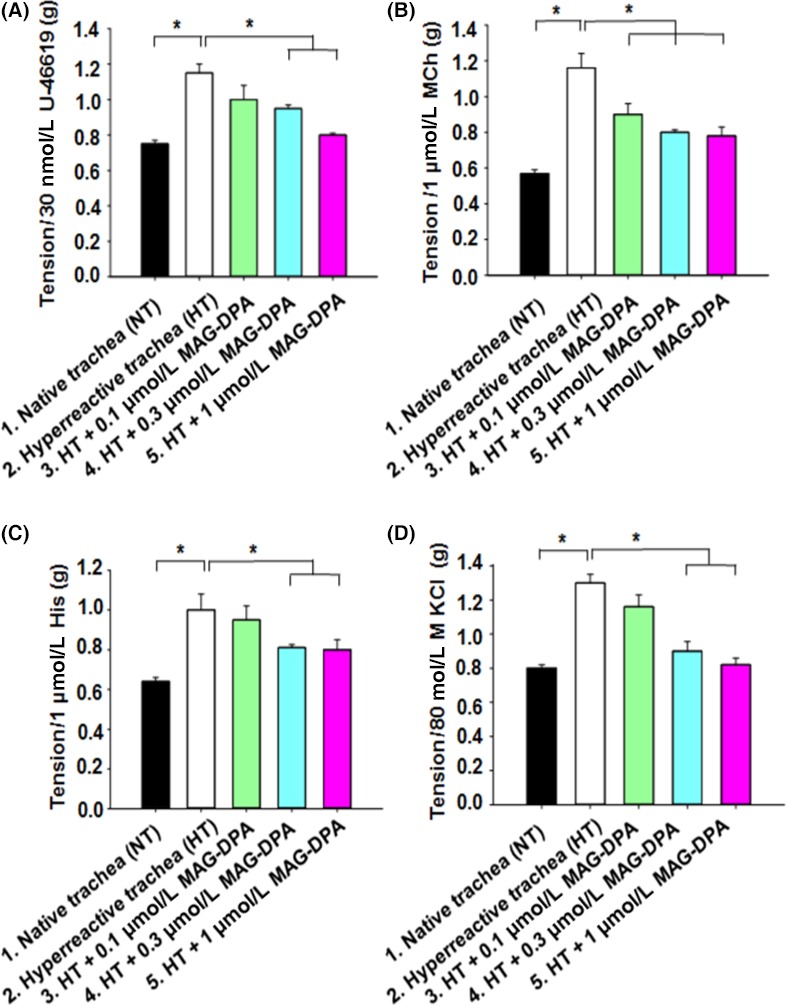
Functional inhibition induced by MAG‐DPA on intrinsic guinea pig tracheal hyperresponsiveness. Bar histogram displaying the mean contractile responses induced by 30 nmol/L U‐46619 (A), 1 *μ*mol/L MCh (B), 1 *μ*mol/L His (C), and 80 mmol/L KCl (D) on native trachea (NT) and hyperreactive trachea (HT). HT were pretreated with either 0.1 *μ*mol/L MAG‐DPA, 0.3 *μ*mol/L MAG‐DPA, or 1 *μ*mol/L MAG‐DPA. Each bar represents the mean amplitude ± SEM with *n* = 12 for each condition (*P ˂ 0.05).

Our group has previously developed and reported an organ culture guinea pig model of AHR (Morin et al. 2005). Hence, we have demonstrated an increased detection of TNF‐*α* in microsomal fractions derived from 72‐h cultured guinea pig tracheas (Khaddaj‐Mallat and Rousseau 2015a). To assess the potential curative effects of MAG‐DPA on pre‐established intrinsic hyperreactivity, pharmacological challenges using 30 nmol/L U‐46619 (Fig. 5A), 1 *μ*mol/L MCh (Fig. 5B), and 1 *μ*mol/L His (Fig. 5C) were performed on guinea pig tracheal rings under the same experimental conditions. Compared to controls (native), hyperresponsive tracheal rings significantly increased the amplitude of the tonic responses to either 30 nmol/L U‐46619, 1 *μ*mol/L MCh, or 1 *μ*mol/L histamine. However, treatments with increasing concentrations of MAG‐DPA were able to significantly blunt this overreactivity, essentially returning the agonist‐induced tone near the tension level developed by the native guinea pig tracheas (Fig. 5D).

In addition, the effects of MAG‐DPA were assessed on AHR in the present guinea pig tracheal model using a depolarizing agent (80 mmol/L KCl). In Figure 5C, native and hyperreactive tracheal rings (following 72 h of organ culture) in the absence or presence of 0.1, 0.3, or 1 *μ*mol/L MAG‐DPA were contracted with 80 mmol/L KCl isosmotic solution. Amplitude of guinea pig tracheal responses confirmed that the cultured hyperresponsive tracheas displayed larger responses than the control tracheas, while MAG‐DPA induced a net concentration‐dependent decrease in the mean contractile responses as compared to the contractile responses from hyperresponsive tracheas.

### Anti‐inflammatory effect of MAG‐DPA on guinea pig trachea and smooth muscle cells

**Figure 6:**
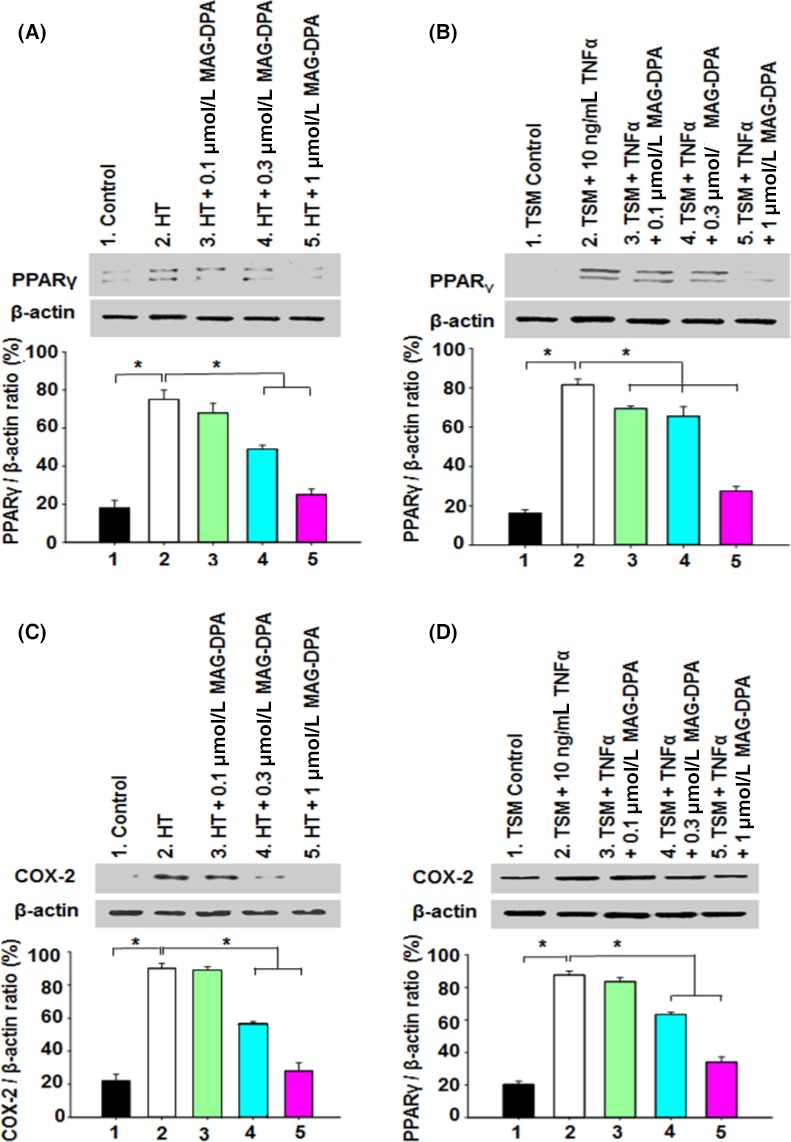
Effect of MAG‐DPA on PPAR *γ* and COX‐2 expression levels in 3‐day cultured guinea pig tracheal tissues and in guinea pig tracheal smooth muscle (TSM) pretreated with TNF‐*α*. (A) Western blot analysis of nuclear protein fractions derived from control, hyperresponsive tracheas (HT), HT + 0.1 *μ*mol/L MAG‐DPA, HT + 0.3 *μ*mol/L MAG‐DPA, and HT + 1 *μ*mol/L MAG‐DPA‐treated rings using specific antibodies against PPAR *γ* and *β*‐actin. Quantitative analysis of PPAR *γ*/*β*‐actin density ratio as a function of experimental conditions (*n* = 6, *P ˂ 0.05). (B) Western blot and quantitative analysis of PPAR *γ*/*β*‐actin density ratio in TSM lysates derived from control, 10 ng/mL TNF‐*α*, TNF‐*α* + 0.1 *μ*mol/L MAG‐DPA, TNF‐*α* + 0.3 *μ*mol/L MAG‐DPA, and TNF‐*α* + 1 *μ*mol/L MAG‐DPA‐treated TSM (*n* = 4, *P ˂ 0.05). (C) Western blot analysis of subcellular fractions derived from control, HT, HT + 0.1 *μ*mol/L MAG‐DPA, HT + 0.3 *μ*mol/L MAG‐DPA, and 1 *μ*mol/L MAG‐DPA‐treated rings using specific antibodies against COX‐2 and *β*‐actin. Quantitative analysis of COX‐2/*β*‐actin density ratio as a function of experimental conditions (*n* = 6, *P ˂ 0.05). (D) Western blot analysis of TSM lysates derived from control, TNF‐*α*, TNF‐*α* + 0.1 *μ*mol/L MAG‐DPA, TNF‐*α* + 0.3 *μ*mol/L MAG‐DPA, and 1 *μ*mol/L MAG‐DPA‐treated TSM using specific antibodies against COX‐2 and *β*‐actin. Quantitative analysis of COX‐2/*β*‐actin density ratio as a function of experimental conditions (*n* = 4, *P ˂ 0.05).

NF*κ*B and PPAR*γ* are the main transcription factors associated with inflammatory status in several tissues, including airways (Christman et al. 2000; Becker et al. 2006). Experiments were designed to determine the detection levels of PPAR*γ* and COX‐2 in control, hyperresponsive tracheas, as well as hyperresponsive tracheas under pretreated experimental conditions with either 0.1, 0.3, or 1 *μ*mol/L MAG‐DPA. Results show that, compared to controls, guinea pig hyperresponsive tracheas significantly increased the staining density of the PPAR*γ* immunoreactive band. However, 1 *μ*mol/L MAG‐DPA induced a significant inhibition of this upregulation induced by the overall intrinsic hyperresponsiveness mechanism. Quantitative results suggest that MAG‐DPA induced a concentration‐dependent decrease in PPAR*γ*/*β*‐actin ratio, as compared to the enhanced ratio detected in guinea pig hyperresponsive trachea (Fig. 6A). A previous study has reported that omega‐3 derivatives such as MAG‐EPA and 17,18‐EpETE mediate proresolving effect on TNF‐*α*‐pretreated tracheal smooth muscle cells by inhibiting I*κ*B*α* degradation (Khaddaj‐Mallat and Rousseau 2015a). Complementary experiments were also performed to assess the status of PPAR*γ* in the guinea pig tracheal smooth muscle (TSM) model of inflammation. Smooth muscle lysates were either untreated (control) or treated with TNF‐*α* (10 ng/mL) in the absence or presence of 0.1, 0.3, or 1 *μ*mol/L MAG‐DPA. Relative detection levels calculated from western blots performed on these preparations revealed that PPAR*γ* was present in all tested lysates, although TNF‐*α* treatment increased the ratio of detection of PPAR*γ*/*β*‐actin (Fig. 6B). A concentration‐dependent decrease in PPAR*γ*/*β*‐actin ratio was measured following MAG‐DPA treatments, compared with the corresponding ratio detected in TNF‐*α* alone.

Finally, the effects of MAG‐DPA were assessed on COX‐2 protein detection immunostaining in nuclear fractions of guinea pig tracheal rings (Fig. 6C) and in isolated guinea pig smooth muscle cell lysates (Fig. 6D) using western blot analysis. Results demonstrate that COX‐2 expression levels were increased in hyperresponsive tracheas (Fig. 6C) as well as in tracheal smooth muscle pretreated with 10 ng/mL TNF‐*α* (Fig. 6D), whereas MAG‐DPA treatments reduced COX‐2 immunostaining levels in a concentration‐dependent manner.

## Discussion

In this study, we investigated the ability of MAG‐DPA to reverse the inflammation and overreactivity triggered by either TNF‐*α* or IL‐13 in human distal bronchi. These observations were also supported in a guinea pig model of organ‐cultured tracheal rings. To our current knowledge, this is the first report directly assessing the mode of action of MAG‐DPA in human and rodent models of airway hyperresponsiveness. DPA monoacylglyceride is known for its antitumorigenic (Morin et al. 2013c) and proresolving properties in pulmonary artery vasculature (Morin et al. 2014). In this study, MAG‐DPA further displayed anti‐inflammatory effects that were amplified in the presence of ASA. These effects were likely related to lower phosphorylation levels of NF*κ*B and CPI‐17, as well as a downregulation in PPAR*γ* and COX‐2 expression in human bronchi treated with TNF‐*α* or IL‐13 and guinea pig tracheal rings.

It was of interest to analyze the acute effect of MAG‐DPA on airway active tone. However, MAG‐DPA had a very low relaxing effect on native tracheal tone triggered by MCh, a muscarinic agonist. This current observation suggests that MAG‐DPA had relatively poor direct bronchorelaxing properties. However, it did not rule out the possibility that it could modulate inflammation and AHR.

Moreover, several cytokines and chemokines are overexpressed in asthmatic airways, resulting in mucus secretion, smooth muscle contraction, and overall bronchial hyperresponsiveness (Olin and Wechsler 2014). In our study, human and guinea pig cultured model were used to facilitate the study of MAG‐DPA effects on the pharmacological and biochemical properties that characterize airway responsiveness. Prior evidences suggest that organ culture of airway tissue maintained the airway morphology and increased the pharmacological reactivity to spasmogens. Nevertheless, cultured explants were associated with structural changes such as the presence of apoptotic bodies in the submucosa and epithelial layers, but none in the smooth muscle layer (Morin et al. 2005). Furthermore, the amplitudes of pharmacological responses to various spasmogens confirmed the key role of proinflammatory cytokines in ASM hyperresponsiveness (Tliba et al. 2003; Morin and Rousseau 2006). For instance, TNF‐*α* is released from airway resident macrophages (Brightling et al. 2008), whereas IL‐13 is secreted by Th_2_ cell‐type responses (Tliba et al. 2003). These proinflammatory cytokines also exert important amplifying effects on inflammatory biomarkers, which were reversed in a concentration‐dependent manner by MAG‐DPA in both mammalian models used in this study. Recently, epithelial cells were found to participate in airway smooth muscle inflammatory reactions by releasing eicosanoids, cytokines, and nitric oxide (Davies 2014; Park et al. 2015). Previous studies have already demonstrated that IL‐13 activates the TNF‐*α*/NF*κ*B pathway in human lung tissues (Chapoval et al. 2007; Khaddaj‐Mallat et al. 2015b), while MAG‐DHA and RvD1 reverses, the IL‐13 stimulates I*κ*B*α* ubiquitination and proteasomal degradation, which in turn alleviates the phosphorylation of the p65‐NF*κ*B subunits (Chapoval et al. 2007; Goto et al. 2009). It has been demonstrated that MAG‐DPA reduces inflammation and proliferation of pulmonary artery smooth muscle cells in a rat model of pulmonary hypertension (Morin et al. 2014). DPA has also been demonstrated to have a potent inhibitory effect on angiogenesis through the suppression of VEGFR‐2 expression in bovine aortic endothelial cells (Tsuji et al. 2003), while oral supplementation with 21.2 mg/kg DPA was found to lead to higher EPA concentrations in the kidney of Sprague Dawley rats (Holub et al. 2011; Kelly et al. 2011). In a previous report, MAG‐EPA treatments were shown to decrease the contractile reactivity and nuclear protein expression in 72‐h cultured and TNF‐*α*‐treated guinea pig tracheal rings (Khaddaj‐Mallat and Rousseau 2015a). In the current study, molecular investigations revealed that MAG‐DPA impedes airway inflammation biomarkers at tissular level by downregulating the phosphorylated form of p65‐NF*κ*B, as well as the COX‐2 expression level in subcellular fractions derived from human bronchi. Previous data demonstrated that the intrinsic overreactivity in cultured guinea pig tracheal rings was associated with an upregulation of TNF‐*α* in the microsomal fractions derived from hyperresponsive guinea pig cultured explants (Khaddaj‐Mallat and Rousseau 2015a). Several studies have already attested that specific transcription factors, such as NF*κ*B and PPAR*γ*, were involved in the regulation of lung inflammatory diseases (Christman et al. 2000; Becker et al. 2006). Hence, current data suggest that PPAR*γ* signaling participates in the anti‐inflammatory effects of MAG‐DPA via a blunting of the NF*κ*B pathway, resulting in the downregulation of proinflammatory gene products. Of interest, the resolving effects of MAG‐DPA, this is sequentially metabolized in DPA and other lipid mediators.

However, little is known regarding the anti‐inflammatory effect of MAG‐omega‐3 compounds in vivo, Morin et al. (2015) have demonstrated that MAG‐DPA downregulated the COX‐2 expression in rheumatoid arthritis model. In addition, MAG‐EPA as well as MAG‐DHA supplementations of ovalbumin‐sensitized guinea pig were able to alleviate the expression levels of proinflammatory cytokines (IL‐13, TNF‐*α*) in an allergic model of asthma (Morin et al. 2012b, 2013a). Specifically, the anti‐inflammatory effects of these compounds in the airways were correlated with reduced goblet cell hyperplasia and mucus production in guinea pig bronchial sections, without any toxic effects on epithelial cells (Morin et al. 2013a).

Asthma is a chronic inflammatory disease in which inflammation causes an associated increase in airway responsiveness to a variety of stimuli (Murdoch and Lloyd 2010). Cytokines and proinflammatory eicosanoids, such as prostaglandins, leukotrienes, and thromboxane, were previously shown to increase the pharmacological reactivity and the Ca^2+^ sensitivity due to an enhanced phosphorylation level of the main regulatory proteins of myofilaments such as CPI‐17 and RhoA in human and rodent ASM (Hunter et al. 2003; Chiba et al. 2010). One of our objectives was to demonstrate the ability of MAG‐DPA to abolish airway hyperresponsiveness induced by short‐term (48 h) TNF‐*α* and IL‐13 pretreatments of human bronchi. The present data also show that MAG‐DPA treatments inhibited the pharmacological responses, thus confirming that submicromolar concentrations of MAG‐DPA were able to induce a similar effect in both models of airway hyperresponsiveness, as well as CPI‐17 phosphorylation in cytosolic fractions derived from IL‐13‐treated human bronchi. Our data also revealed that following 1 *μ*mol/L MAG‐DPA treatment, the mechanical reactivity of hyperresponsive tracheal rings to various spasmogens were maintained above the level recorded under control conditions in native trachea, while MAG‐DPA normalized the response to 80 mmol/L KCl, suggesting a significant effect on Ca^2+^ signaling. Moreover, it is widely accepted that long‐term cytokine treatments induce Ca^2+^ hypersensitivity in human ASM (Hunter et al. 2003; Morin and Rousseau 2006; Khaddaj‐Mallat et al. 2015b). Our current data reveal that 1 *μ*mol/L MAG‐DPA reversed the Ca^2+^ sensitivity induced by TNF‐*α* due to a decrease in CPI‐17 phosphorylation levels in human bronchi. This reduction in CPI‐17 phosphorylation level in pathological human lungs through the use of MAG‐DPA could prove highly relevant in improving the respiratory capacities of patients with inflammatory respiratory diseases such as asthma and COPD. Moreover, it would be important to test the effect MAG‐DPA on Rho‐kinase pathway which is implicated in the regulation of Ca^2+^ sensitivity of ASM (Hunter et al. 2003).

It was also of prime interest to identify a specific compound that could significantly oppose the inflammatory status and would result in a decrease in airway hyperresponsiveness induced by various proinflammatory cytokines or culture of native guinea pig tracheal explants. Various studies have reported conflicting data regarding the clinical value of *n*−3 polyunsaturated fatty acid in the treatment of asthma (Reisman et al. 2006; Brannan et al. 2015). These studies reported a decrease in activation of immune cells without changing the severity of asthma. Hence, the absorption rate of free PUFAs, as negatively charged lipids, was not tested in these reports. In contrast, previous studies have demonstrated that omega‐3 fatty acids in monoacylglyceride form displayed an increased systemic bioavailability in an allergic model of asthma and in a xenograft mice model of lung cancer, likely due to an increased absorption rate, which in turn could be transformed into antiphlogistic lipid mediators, such as resolvins (Morin et al. 2012b; Morin et al. 2013a,b).

Several reports have highlighted the role of aspirin (ASA) in combination with omega‐3 fatty acids on the resolution of inflammatory signaling pathways (Blocker et al. 2012; Khaddaj‐Mallat et al. 2015b). Blocker et al. (2012) demonstrated that EPA + DHA combined to aspirin were able to trigger anti‐inflammatory and antiangiogenesis effects in healthy adults. In this study, results clearly demonstrate that contractile responses and CPI‐17 phosphorylation were all decreased in IL‐13‐treated human bronchi following MAG‐DPA and ASA treatments. The present findings warrant further studies to investigate the combined role of MAG‐DPA plus ASA on cytokine‐mediated inflammatory biomarkers expression.

### Study limitations

The ability of MAG‐DPA to generate new proresolving mediators that would act as agonists on specific receptors GPR32, ChemR23, and ALX/FPR2 (Serhan et al. 2000; Li et al. 2013) have not been directly assessed in our study. Complementary data (Plate S1 and 2) demonstrated that the GPR‐32 receptor was downregulated by TNF‐*α* or IL‐13, while being upregulated by MAG‐DPA, MAG‐DPA combined to ASA, and RvD1. RvD1 is a putative DHA metabolite, which was described as a potent proresolving compound (Serhan et al. 2000). Thus, further biochemical analysis would be requested to measure the ability of MAG‐DPA to generate these proresolving mediators.

In summary, to our knowledge, this is the first study demonstrating that MAG‐DPA interacts with TNF‐*α*/NF*κ*B and PPAR*γ* signaling pathways to mediate resolving properties in human and rodent models of airway hyperresponsiveness. In addition, the present findings demonstrate that MAG‐DPA is able to decrease the activation of PKC/CPI‐17 pathway, leading to a rightward shift in Ca^2+^ sensitivity and a significant decrease in airway hyperresponsiveness. The clinical relevance of MAG‐DPA would be related to its capacity to generate lipid metabolites, such as Resolvin D1, which was associated with a concomitant increase in GPR‐32 expression. The ability of the Resolvin precursor to reduce both the inflammation and hyperresponsiveness of airway smooth muscle could be of valuable pathophysiological significance in the treatment of respiratory diseases such as asthma, COPD, and cystic fibrosis.

## Disclosure

None of the authors has a financial relationship with a commercial entity that has an interest in the subject of this manuscript.

## Supporting information


**Plate S1.** Pharmacological effects of MAG‐DPA on GPR‐32 protein expression in TNF‐*α*‐pretreated human bronchi.
**Plate S2.** Effects of MAG‐DPA on GPR‐32 protein expression in IL‐13‐pretreated human bronchi.
