# Supplementary material for: MAG‐DPA curbs inflammatory biomarkers and pharmacological reactivity in cytokine‐triggered hyperresponsive airway models
Source: Pharmacol Res Perspect. 2016 Oct 18;4(6):e00263. doi: 10.1002/prp2.263 (PMC5226286; doi:10.1002/prp2.263)
Supplement: Supplementary file 1 — Plate S1. Pharmacological effects of MAG‐DPA on GPR‐32 protein expression in TNF‐α‐pretreated human bronchi. Plate S2. Effects of MAG‐DPA on GPR‐32 protein expression in IL‐13‐pretreated human bronchi. [file PRP2-4-e00263-s001.pdf]

Supplementary material

Plate 1

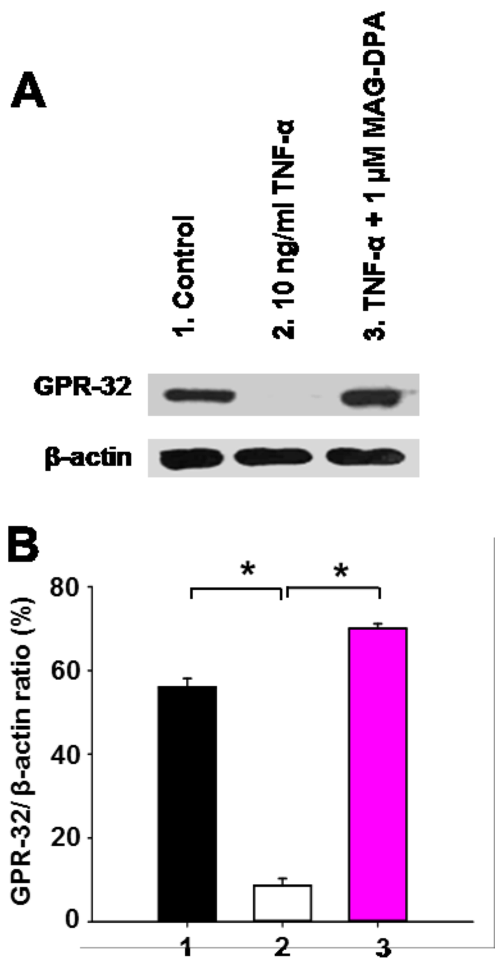

Plate 1 : Pharmacological effects of MAG-DPA on GPR-32 protein expression in TNF-α-pretreated human bronchi. (**A**) Microsomal fractions derived from control or 10 ng/ml TNF-α treated bronchi in the absence or presence of 1 µM MAG-DPA were stained using specific primary antibodies against GPR-32 and β-actin and selective secondary antibobyies.
(**B**) Quantitative analyses of GPR-32 density ratio. Staining densities of GPR-32 were expressed as a function of β-actin signals (n = 3, *$P < 0.05$).

Supplementary material

Plate 2

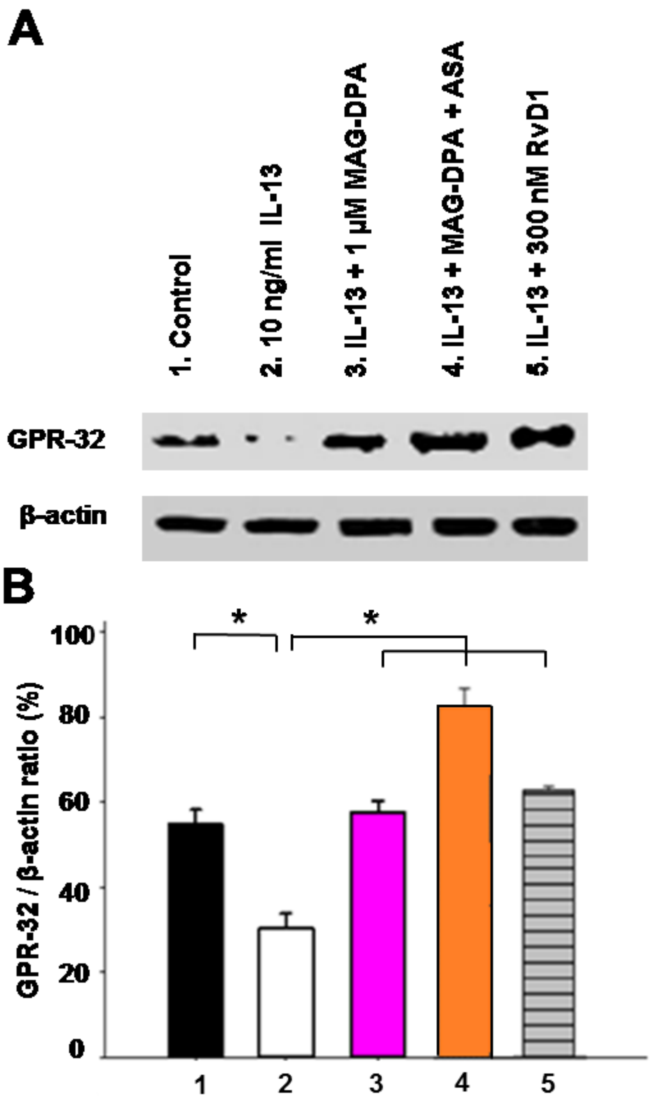

Plate 2 : Effects of MAG-DPA on GPR-32 protein expression in IL-13-pretreated human bronchi. (A) Western blot and quantitative analysis of GPR-32 density ratios in microsomal fractions derived from human bronchi obtained from control (untreated), 10 ng/ml IL-13, IL-13 + 1 µM MAG-DPA, IL-13 + MAG-DPA + 100 µM ASA and IL-13 + 300 nM RvD1-treated human bronchi. **(B)** Quantitative analysis of GPR32/ β-actin density ratios. Staining densities in human bronchial protein fractions are expressed as a function of β-actin signals (n = 6, *$P < 0.05$).
